# The Same Metabolic Response to FGF21 Administration in Male and Female Obese Mice Is Accompanied by Sex-Specific Changes in Adipose Tissue Gene Expression

**DOI:** 10.3390/ijms221910561

**Published:** 2021-09-29

**Authors:** Elena Makarova, Antonina Kazantseva, Anastasia Dubinina, Tatiana Jakovleva, Natalia Balybina, Konstantin Baranov, Nadezhda Bazhan

**Affiliations:** 1 The Laboratory of Physiological Genetics, The Institute of Cytology and Genetics, 630090 Novosibirsk, Russia; antonyna-sh@yandex.ru (A.K.); dubinina_anastas@mail.ru (A.D.); tatyanajakovleva@yandex.ru (T.J.); n.balybina@alumni.nsu.ru (N.B.); bazhan-nm@yandex.ru (N.B.); 2 The Institute of Molecular and Cellular Biology, 630090 Novosibirsk, Russia; baranov@mcb.nsc.ru

**Keywords:** FGF21, mice, obesity, food preferences, sex differences, gene expression

## Abstract

The preference for high-calorie foods depends on sex and contributes to obesity development. Fibroblast growth factor 21 (FGF21) beneficially affects taste preferences and obesity, but its action has mainly been studied in males. The aim of this study was to compare the effects of FGF21 on food preferences and glucose and lipid metabolism in C57Bl/6J male and female mice with diet-induced obesity. Mice were injected with FGF21 or vehicle for 7 days. Body weight, choice between standard (SD) and high-fat (HFD) diets, blood parameters, and gene expression in white (WAT) and brown (BAT) adipose tissues, liver, muscles, and the hypothalamus were assessed. Compared to males, females had a greater preference for HFD; less WAT; lower levels of cholesterol, glucose, and insulin; and higher expression of *Fgf21*, *Insr*, *Ppara*, *Pgc1*, *Acca* and *Accb* in the liver and *Dio2* in BAT. FGF21 administration decreased adiposity; blood levels of cholesterol, glucose, and insulin; hypothalamic *Agrp* expression, increased SD intake, decreased HFD intake independently of sex, and increased WAT expression of *Pparg, Lpl* and *Lipe* only in females. Thus, FGF21 administration beneficially affected mice of both sexes despite obesity-associated sex differences in metabolic characteristics, and it induced female-specific activation of gene expression in WAT.

## 1. Introduction

Obesity is a serious problem in modern society. Over the past few decades, the main factor contributing to the development of obesity has been overeating [1]. The preference for palatable, high-calorie food induces overeating and contributes to the epidemic prevalence of obesity [2,3]. In this regard, it is of particular interest to search for drugs that not only correct metabolic disorders associated with obesity but also shift taste preferences toward food products with a balanced composition of macronutrients.

Fibroblast growth factor 21 (FGF21) is purported to be one of the most promising candidates for the development of such drugs. FGF21 is synthesized and secreted into the circulation mainly by the liver in response to metabolic stresses. It functions to restore homeostasis by coordinating metabolic responses from brown and white adipose tissues, muscles, and liver to these stressors [4], and by modifying eating behavior by influencing taste preferences [5].

FGF21 has potent beneficial effects on obesity and diabetes which have been observed in rodents, monkeys, and humans [6]. In mice, administration of FGF21 reduces body weight, increases insulin sensitivity, normalizes blood glucose levels [7,8,9,10], increases protein intake [11], and reduces sugar intake [12].

The ability of FGF21 to promote healthy macronutritional intake increases its attractiveness for the complex therapy of metabolic disorders. Both sugars and fats are very appealing, and FGF21 was shown to decrease the intake of sugars but not fats [13]. However, genetic studies in humans have revealed an association between single-nucleotide polymorphisms in the locus encoding FGF21, increased intake of sugars, and decreased intake of both proteins and fats [14,15,16]. These data suggest that FGF21 may influence cravings for fat consumption.

FGF21-induced changes in sugar and protein consumption are generally not accompanied by changes in total energy intake [5,11]. This suggests that the regulatory response to FGF21 administration involves the central mechanisms underlying the maintenance of energy homeostasis, including the melanocortin system of the hypothalamus, which regulates food intake and energy expenditure [17]. However, the response of the central melanocortin system to FGF21 administration has not been well studied.

Currently, intensive research is underway to create drugs based on FGF21 [18,19]. However, preclinical studies of the action of FGF21, its analogs, and its mimetics have been carried out mainly on males [7,8,10,20,21]. According to the recommendations of the National Institutes of Health, sex should be recognized as an important biological variable that must be considered when conducting preclinical studies [22,23]. In a few studies performed on rats and mice of both sexes, some effects of FGF21 have been found to be sex-dependent. Sex-based differences in the expression of FGF21 in the liver and other tissues have been found [24,25,26,27], which exhibited differential manifestation with respect to obesity and starvation [28]. Transgenic overexpression of FGF21 had different effects on certain metabolic and hormonal parameters depending on sex [29]. In *A^y^* mice with genetically induced obesity, females, unlike males, were resistant to the catabolic effects of FGF21 [30]. These data suggest that the physiological and pharmacological effects of FGF21 may vary among individuals of different sexes.

The aim of this study was to assess whether the effects of FGF21 administration on glucose and fat metabolism, choice between standard and high-fat diets, and the expression of hypothalamic neuropeptides regulating food intake depend on sex in mice with diet-induced obesity. We found that the metabolic response to FGF21 administration in males and females was similar, despite sex differences in metabolic characteristics and the expression of certain genes in the liver and adipose tissues. In both males and females, administration of FGF21 shifted food choice toward a standard diet, but this response was more pronounced in males. The same beneficial metabolic outcomes of FGF21 administration in males and females were associated with the activation of gene expression of PPARg and its target genes in abdominal adipose tissue only in females.

## 2. Results

### 2.1. Body Weight, Food Intake and Locomotor Activity

At the start of the experiment, male mice were more obese than female mice: they had more absolute and relative fat (Table 1). However, there were no sex differences in the relative weight of interscapular BAT (iBAT) (Table 1).

**Figure 1:**
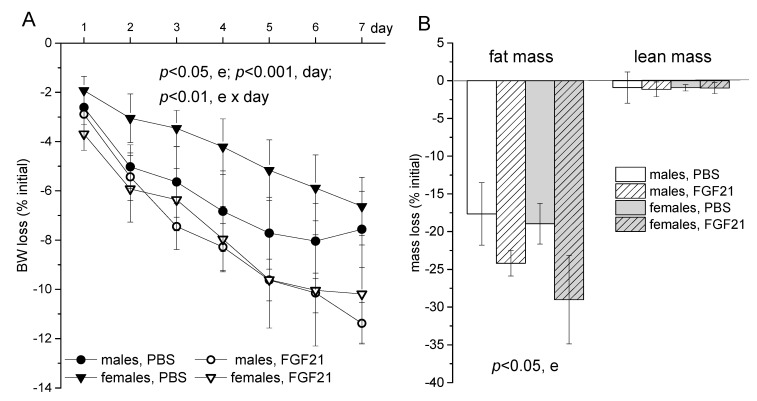
Influence of FGF21 administration on BW (**A**) and lean and fat masses (**B**) in obese male and female C57Bl mice. Data are presented as mean ± SEM. Mice were administered FGF21 for 7 days, and weight loss was calculated as % of initial BW. A: significant influence of factor “e” (experiment: administration of PBS or FGF21), “day” (7), and e × day, repeated measures ANOVA; B: significant influence of factor “e”, two-way ANOVA with factors “experiment” and “sex”.

Injections of both PBS and FGF21 induced weight loss in both males and females. The lowering of BW in the control groups suggests that the mice were stressed by their relocation to new cages and the daily injections. However, weight loss was more pronounced in groups that received FGF21 compared to control groups (*p* < 0.05, factor “experiment”; *p* < 0.01, interaction “experiment” × “day of experiment”; repeated measures ANOVA, Figure 1A). Weight decreased due to the loss of fat mass, and lean mass did not change during the experiment in any group. FGF21 administration intensified fat loss (*p* < 0.05, factor “experiment”; two-way ANOVA with factors “sex” and “experiment”, Figure 1B).

**Figure 2:**
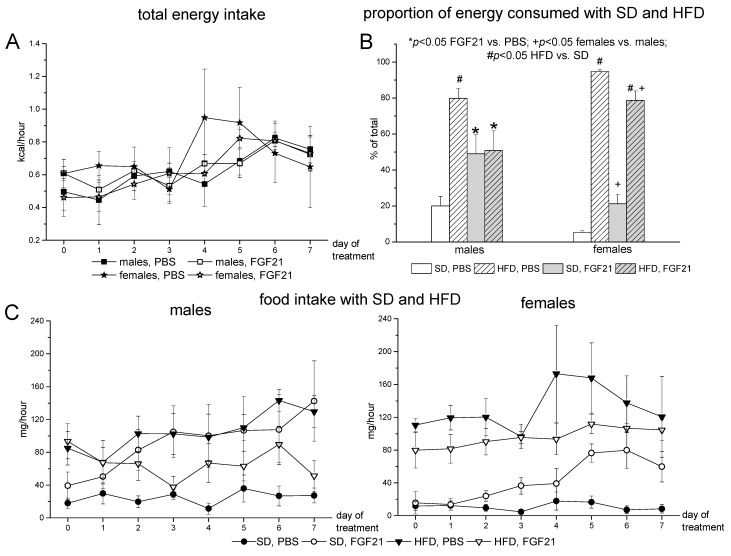
Influence of FGF21 administration on total energy intake (**A**), proportion of energy consumed by SD or HFD (**B**), and food intake with SD or HFD (**C**) in male and female obese mice. Data are presented as mean ± SEM. Male and female mice were fed both pelleted SD and HFD and were administered FGF21 or PBS for 7 days. Intake of energy (kcal/h) and food (mg/h) was calculated as intake per hour on average per day. The share of energy consumed by standard or high-fat diets was calculated as the 7-day average for each mouse. (**A**): *p* < 0.001, day of experiment, repeated measures ANOVA; (**B**): * *p* < 0.05, FGF21 vs. PBS; + *p* < 0.05, females vs. males; # *p* < 0.05, HFD vs. SD, Newman–Keuls post hoc test, three-way ANOVA with factors “experiment” (FGF21, PBS), “sex”, and “diet” (SD, HFD).

The BW decrease was not accompanied by a reduction in energy intake. On the contrary, energy intake increased over the course of the experiment (repeated measures ANOVA, *p* < 0.001, day of experiment, Figure 2A). The total energy intake was the same in males and females; however, the ratio of energy intake/BW was higher in females than in males (repeated measures ANOVA, *p* < 0.05, sex, data are not shown). FGF21 administration did not affect total energy intake, but did affect taste preferences. In the control groups, both males and females preferred HFD, and mice of both sexes consumed a significantly higher proportion of energy by HFD (Figure 2B) and HF chow (Figure 2C). Females preferred FHD more than males, and the proportion of energy intake by HFD was higher in females than in males (repeated measures ANOVA, *p* < 0.05, sex). FGF21 administration increased the consumption of SD and decreased the consumption of HFD, and males were more sensitive to this influence of FGF21. In FGF21-treated males, the contribution of SD and HFD to total energy intake was the same (Figure 2B), and the consumption of standard chow was higher than the consumption of HF chow (Figure 2C). In females, FGF21 administration only reduced the preference for fatty food, but the FGF21-induced changes in the consumption of SD and HFD were not statistically significant (Figure 2B). FGF21 administration changed taste preferences only in males, and as a result, sex differences were observed in the consumption of different kinds of food in FGF21-treated mice (Figure 2B).

There were no sex differences in locomotor activity, and this variable was not affected by FGF21 administration (data not shown).

### 2.2. Blood Parameters

Males and females did not differ in their concentrations of free fatty acids (FFA) and triglycerides in the blood. FGF21 administration did not affect these parameters (Table 2). Blood concentrations of cholesterol, glucose, and insulin were higher in males in both PBS- and FGF21-treated groups. In both males and females, FGF21 administration decreased blood concentrations of cholesterol, glucose, and insulin. Sex differences in blood concentrations of adiponectin and leptin were observed; adiponectin levels were higher in females, and leptin levels were higher in males. FGF21 administration did not affect the levels of these hormones in the blood (Table 2).

### 2.3. Gene Expression

**Figure 3:**
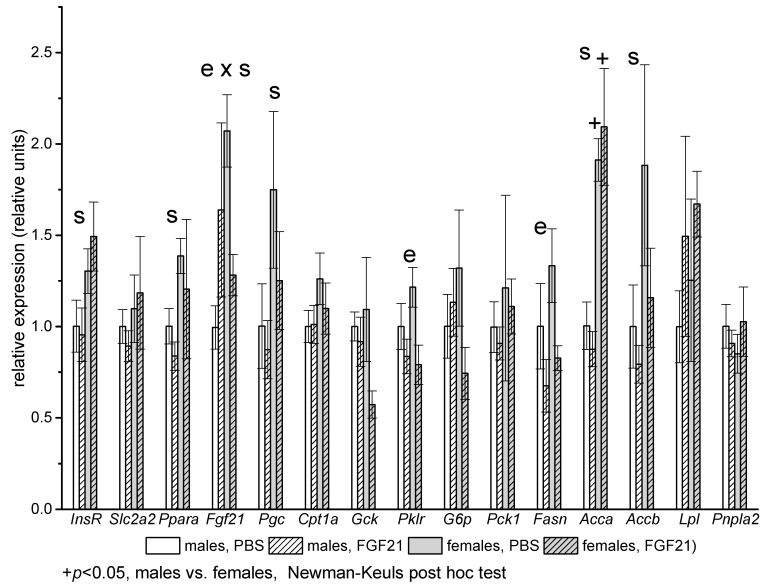
Influence of FGF21 administration on liver gene expression in male and female obese mice. Data are presented as mean ± SEM. Significant influence (*p* < 0.05) of factors “e” (administration of PBS or FGF21), “s” (sex), and “e × s”, two-way ANOVA, are indicated in the plot.

In the liver, sex differences in the expression of genes related to insulin action (insulin receptor, *Insr*), lipid oxidation (transcription factor PPARa, *Ppara*; transcriptional coactivator PGC, *Pgc1*), and lipogenesis (acetyl-CoA carboxylases *Acca* and *Accb*) were observed. For all genes, mRNA levels were higher in females than in males. FGF21 administration decreased the mRNA levels of genes encoding the glycolytic enzyme pyruvate kinase *(Pklr*) and the lipogenesis enzyme fatty acid synthase (*Fasn)* in both males and females. A significant interaction between the factors “sex” and “experiment” was detected for mRNA levels of FGF21. In PBS-treated mice, FGF21 mRNA levels were significantly higher in females (*p* < 0.001, Student’s *t*-test). Administration of FGF21 decreased FGF21 mRNA levels in females and eliminated these differences (Figure 3).

**Figure 4:**
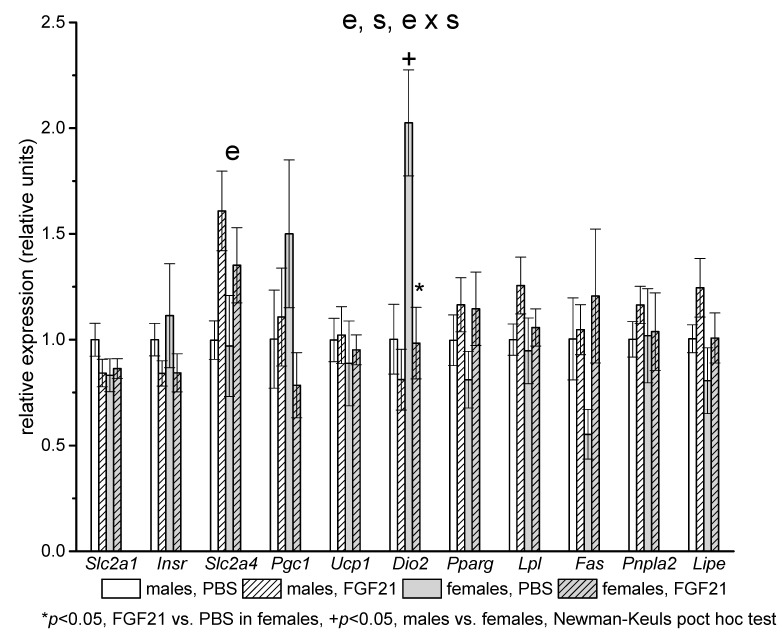
Influence of FGF21 administration on iBAT gene expression in male and female obese mice. Data are presented as mean ± SEM. Mice were administered FGF21 for 7 days. Significant influences of factor “e” (administration of PBS or FGF21), “s” (sex), and “e × s”, two-way ANOVA, are indicated in the plot.

In iBAT, sex differences were observed in the expression of the gene encoding iodothyronine deiodinase (*Dio2*), which was two times higher in females than in males in the control groups. FGF21 administration decreased *Dio2* expression in females, and eliminated sex differences in the expression of this gene (Figure 4). FGF21 administration increased mRNA levels of the gene encoding insulin-regulated glucose transporter 4 (*Slc2a4*) in both males and females (Figure 4).

**Figure 5:**
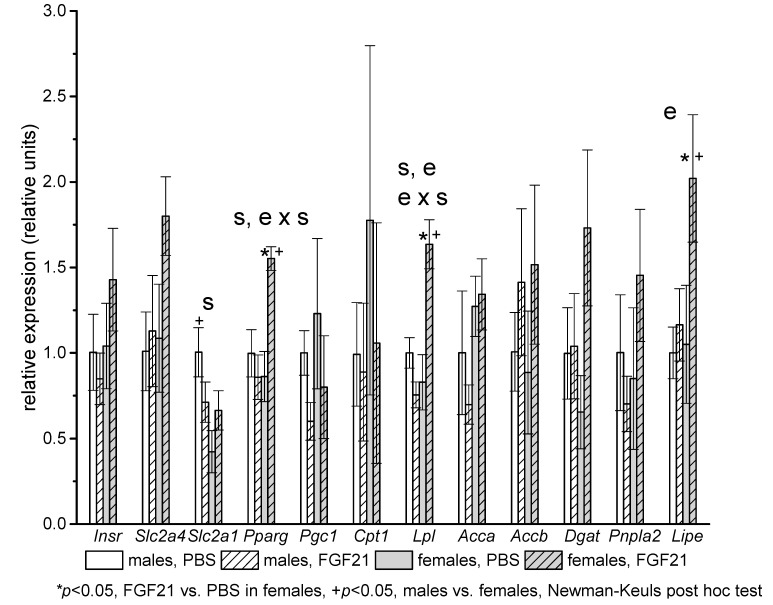
Influence of FGF21 administration on gene expression in abdominal WAT in male and female obese mice. Data are presented as mean ± SEM. Mice were administered FGF21 for 7 days. Significant influences of factor “e” (administration of PBS or FGF21), “s” (sex), and “e × s”, two-way ANOVA, are indicated in the plot.

In abdominal fat, the gene expression of glucose transporter 1 (*Slc2a1*) was lower in females than in males in the control groups, but FGF21 administration eliminated this difference (Figure 5). FGF21 had sex-specific effects on gene expression in abdominal fat: it increased the expression of genes encoding PPARg, lipoprotein lipase (*Lpl*), and hormone-sensitive lipase (*Lipe*) only in females. Moreover, the mRNA levels of these genes in FGF21-treated mice, in contrast to PBS-treated mice, were higher in females than in males (Figure 5).

There were no sex differences in the expression of genes measured in the muscles (*Cpt1*, *Slc2a4*, *Pgc1a, Ucp1*, *Ucp3*, and *Lipe*). FGF21 administration did not affect the expression of these genes in the muscles.

**Figure 6:**
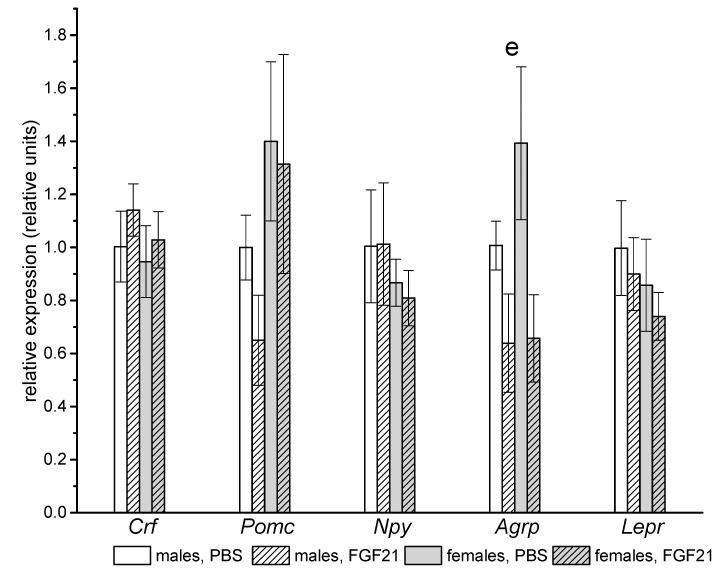
Influence of FGF21 administration on hypothalamic gene expression in male and female obese mice. Data are presented as mean ± SEM. Mice were administered FGF21 for 7 days. Significant influence of factor “e” (administration of PBS or FGF21), two-way ANOVA, is indicated in the plot.

There were no significant sex differences in hypothalamic gene expression. FGF21 administration decreased the gene expression of the orexigenic neuropeptide Agouti-related protein (*Agrp)* in both males and females (Figure 6).

## 3. Discussion

In this study, we evaluated the effect of FGF21 on the metabolism and taste preferences of both male and female mice with diet-induced obesity. Previously, in studies carried out mainly on males, it was shown that both the physiological and pharmacological actions of FGF21 depend on the metabolic state of the individuals. A single administration of FGF21 reduced glucose levels and increased insulin sensitivity in insulin-resistant obese mice [31], whereas it had no detectable effects on metabolism when administered to lean, metabolically healthy animals [32,33]. In mice, sex has a significant impact on food choice [34] and the metabolic response to obesogenic diet consumption [35,36]. Estrogens were shown to protect female mice from obesity and insulin resistance [37,38]. In this regard, we hypothesized that the effect of FGF21 on both metabolic parameters and taste preferences may differ between male and female mice that consumed HFD. We tested this hypothesis in the present work.

In this study, obesity in mice was induced by feeding with a mixture of high-fat and standard diets. This experimental design was chosen in order to avoid the effect of dietary changes (the introduction of a standard diet in addition to a high-fat diet) on animal metabolism when assessing the effect of FGF21 on taste preferences. In these conditions, mice consumed more energy in the HFD, and females preferred the HFD more than males. In addition, females consumed more energy per unit of body weight than males. At the same time, females developed less severe obesity than males, as their absolute and relative amounts of fat were lower than that of males. Sex differences in the adiposity of mice were associated with differences in the hormonal and metabolic parameters of the blood and in the gene expression in the liver and brown fat. Decreased blood glucose and insulin levels and increased liver expression of the insulin receptor gene in females indicated that females were more sensitive to insulin than males. Similar results were obtained by other authors assessing the effect of HFD and the impact of sex on metabolic variables in mice [36,39,40].

The decreased level of leptin in the blood in females compared to males is apparently explained by differences in the amounts of fat mass. Since females consumed more energy per unit of body weight than males and did not differ from males in physical activity, it can be assumed that their lower degree of obesity is explained by higher energy expenditure and greater utilization of fats. This assumption is consistent with the fact that the level of adiponectin and the expression of genes encoding PPARa, FGF21, and PGC1 in the liver and DIO2 in brown fat were higher in females than in males. DIO2 catalyzes the conversion of T4 to its active form, T3, which stimulates fatty acid oxidation and mitochondrial respiration in BAT [41]. Sex differences in energy expenditure can be explained by the inducing effect of estrogens on BAT activity and thermogenesis [42]. The increased expression of *Ppara*, *Fgf21*, and *Pgc1* in the liver, along with an increased level of adiponectin in the blood, indicates the more intensive oxidation of fats in the liver in females than in males, since PPARa [43], PGC1 [44], FGF21, and adiponectin activate fatty acid oxidation in the liver [45].

It is noteworthy that, in this experiment, the level of FGF21 mRNA in the liver in females was twice as high as that in males. Previously, in mice that consumed HFD, we and other authors obtained the opposite result: FGF21 gene expression was higher in males in both obese [28] and nonobese mice [26]. These conflicting shifts in the manifestation of sexual dimorphism in FGF21 gene expression are possibly due to diet composition. Increased *Fgf21* expression in males compared to females was observed when mice consumed a cafeteria diet that mimics the human Western diet [26,28], and it was higher in females when mice consumed a mixture of SD and HFD in the present experiment. The expression of the FGF21 gene in the liver strongly depends on the macronutrient composition of the diet [46]. Previously, we showed that the upregulation of *Fgf21* expression in the liver was biased differently depending on sex and metabolic situation: toward females during adaptation to fasting, but toward males during adaptation to diet-induced obesity [28]. Together, these data suggest that the impact of sex on liver *Fgf21* expression depends not only on the metabolic state but also on the nutrient composition of the diet.

Increased expression of the gene encoding FGF21 in females possibly contributed to their lower susceptibility to the development of obesity, insulin resistance, and hypercholesterolemia, since FGF21 increases energy expenditure through the activation of thermogenesis in BAT [47], activates fatty acid oxidation in the liver [48], increases insulin sensitivity [31], and helps to reduce blood cholesterol levels [49].

Despite the fact that females differed from males in degree of obesity and consumption of a high-fat diet, sex did not influence the pharmacological action of FGF21. In females, as in males, administration of FGF21 reduced body weight and blood levels of cholesterol, glucose, and insulin. FGF21 can induce weight loss in different ways: by increasing energy expenditure through thermogenesis, by increasing physical activity, and, in some cases, by reducing food intake [50]. In this experiment, a decrease in fat mass was associated with an increase in energy expenditure, because energy consumption did not decrease but rather increased during the experiment, and FGF21 did not affect locomotor activity.

The unidirectional beneficial effects of exogenous FGF21 on metabolism in male and female mice with dietary-induced obesity were accompanied by similar changes in the expression of certain genes in the liver and sex-specific changes in gene expression in adipose tissue. Thus, although metabolic outcomes associated with FGF21 administration were equivalent in males and females, some molecular mechanisms underlying FGF21 actions were different between mice of different sexes.

The decrease in blood glucose and insulin levels under the influence of FGF21 indicates an increase in insulin sensitivity. These changes were associated with enhanced expression of the gene encoding GLUT4 in BAT in both male and female mice. These data are in line with the observation that FGF21 substantially enhances insulin sensitivity through its actions on brown adipose tissue [51]. A decrease in the liver expression of genes encoding fatty acid synthase and pyruvate kinase is apparently a consequence of a decrease in the concentrations of insulin and glucose in the blood, since insulin stimulates the expression of the FAS gene [52], and a decrease in insulin and glucose in the blood will promote the activation of gluconeogenesis and a decrease in the rate of glycolysis.

Unlike the liver, adipose tissue is a direct target of the action of FGF21 [51], and it is in adipose tissue that we found a sex-specific response to FGF21 administration in the expression of genes related to lipid metabolism. FGF21 administration decreased the expression of *Dio2* in BAT and increased the expression of genes encoding PPARg, LPL, and HSL in the abdominal WAT, but only in females. The detected changes in gene expression in WAT indicate an intensification of the rate of fat metabolism. PPARg was shown to upregulate preadipocyte differentiation and expression of the HSL gene in differentiating preadipocytes [53], and to increase FFA disposal in the WAT via activation of LPL expression and promotion of triglyceride synthesis in adipocytes [54]. The simultaneous activation of lipogenesis (synthesis of TG) and lipolysis may be one of the mechanisms by which energy expenditure is increased in adipose tissue in females.

In this work, we show for the first time that in females, as in males, FGF21 shifted taste preferences toward a balanced diet versus a high-fat diet, but this effect was less pronounced in females. It is well documented that FGF21 reduces the consumption of sugars: this has been shown not only in males [5,12,13,55], but also in females [56]. Studies on the effect of FGF21 on protein and fat intake have been conducted only in males, and the results characterizing the effect of FGF21 on protein intake are controversial: some studies have shown that FGF21 increases protein intake [11,57], but others have not observed this effect [58]. The authors explain these contradictions by the use of different approaches to assess taste preferences [58]. Results from two studies that assessed the effect of FGF21 on fat intake led the authors to conclude that FGF21 does not affect fat intake [5,11]. In our experiment, the administration of FGF21 significantly reduced the intake of a high-fat diet, possibly due to suppression of the desire to eat fatty foods. This assumption is supported by the fact that a decrease in HFD consumption was accompanied by a decrease in *Agrp* expression in the hypothalamus. AGRP was shown to increase fat consumption via interaction with opioid receptors [59], and hypothalamic opioid receptor systems play an important role in the stimulation of fat intake [60]. The discrepancies in the interpretation of the results concerning the effect of FGF21 on fat consumption between us and other authors are also apparently related to the different designs of the experiments. FGF21 had no effect on the intake of an aqueous emulsion of vegetable oil in a two-bottle test [5] or on the preference between low-fat and high-fat diets containing an equivalent percentage of proteins [11]. The usage of the Geometric Framework revealed that the elevation of FGF21 and its metabolic effects are highly dependent on nutritional context [46]. It can be assumed that FGF21 is able to adapt its action on taste preferences to nutritional conditions in accordance with urgent needs for nutrients and to shift food choice toward a nutrient-balanced diet.

Sex differences in FGF21 liver gene expression and its sex-specific effect on food choice and gene expression in WAT indicate that sex steroids can modulate both the physiological and pharmacological actions of FGF21. To date, the influence of sex steroids on FGF21 functions has not been studied. It was shown that estradiol activates the expression of FGF21 in the mouse liver [61,62], but whether this effect depends on the metabolic status and nutritional composition of food has not been studied. To understand the role of FGF21 in the formation of sex differences in taste preferences and adaptation to the consumption of high-calorie foods, more research is needed on the interactions between FGF21 and sex steroid actions on the central and peripheral regulatory pathways that determine food choice and energy exchange.

A principal limitation of our study is that FGF21 actions may be different depending on species and cannot be directly translated to humans. Additional investigations on the sex-specific actions of FGF21 should be conducted in other preclinical models. In addition, age differences among the mice used in the experiment and small sample sizes (*n* = 5–7) may increase variability in the parameters studied and contribute to the differences observed between groups.

## 4. Materials and Methods

### 4.1. Animals and Experimental Design

C57BL/6J mice were bred in the SPF vivarium of the Institute of Cytology and Genetics. The mice were housed individually under a 13-h/11-h light/dark regime (light from 01:30 to 14:30) at an ambient temperature of 22–24°C. Table 3 illustrates the experimental design. To induce obesity, 17 male and 20 female mice were provided ad libitum access to both standard (SD) and high-fat (HFD) diets and water from the age of 12 weeks. Mice were weighed regularly from the age of 15 weeks, when the average weight of males was 29.4 ± 0.6 (25–36 g), and the average weight of females was 21.4 ± 0.4 (18–24 g). From 16 to 26 weeks of HFD consumption (28–38 weeks of age), the mice with the highest body weight (males, 44–46 g; females, over 30 g) were selected for the experiment and randomly divided into control (administration of PBS) and experimental (administration of FGF21) groups. There were 7 males and 5 females in the control groups and 7 males and 5 females in the experimental groups. Obese mice were placed in PhenoMaster cages (TSE system). The mice were acclimated to the new cages for 2 days. Mouse fat and lean masses were assessed on the third day (day 0), and the injections were started on the fourth day (day 1). Mouse recombinant FGF21 (1 mg per 1 kg) dissolved in PBS or PBS alone were administered subcutaneously at the end of the light period (12:00–13:00) for 7 days.

The day after the last injection, fat and lean masses were measured again, and then the animals were sacrificed by decapitation. Samples of trunk blood were collected, and the liver and interscapular brown adipose tissues (iBAT) were weighed. The samples of the liver, abdominal white adipose tissue (aWAT) in the paragonadal region, subcutaneous white adipose tissue (sWAT) in the inguinal region, iBAT, and skeletal muscle were collected and snap-frozen in liquid nitrogen to evaluate gene expression.

To discriminate between consumption of SD and HFD, the PhenoMaster cages that housed the mice were equipped with two feeding sensors that measures the amount of food consumed over time. BW was measured daily, and intake of standard and high-fat food and locomotor activity were monitored throughout the experimental period. The amount of energy consumed by SD or HFD, the amount of energy consumed by both diets (total energy), and the percentage of energy consumed by SD or HFD in relation to total energy were calculated. Fat and lean masses were measured using an EchoMRI-700 magnetic resonance analyzer (EchoMRI LLC, Houston, TX, USA).

### 4.2. Diets

Standard diet: pelleted Ssniff Rat/Mouse Maintenance diet with metabolizable energy of 3230 kcal/kg (9% from fat, 24% from proteins, and 67% from carbohydrates). High-fat diet: pelleted Ssniff DIO-60kJ fat (Lard) that corresponds to D12492 with metabolizable energy of 5150 kcal/kg (60% from fat, 20% from protein, and 20% from carbohydrates) received from Ssniff Spezialdiäten GmbHFerdinand-Gabriel-Weg D-59494 Soest, Germany.

### 4.3. Plasma Assays

Concentrations of insulin, leptin, and adiponectin were measured using a Rat/Mouse insulin ELISA Kit, a Mouse leptin ELISA Kit (EMD Millipore, St. Charles, MO, USA), and a Mouse adiponectin ELISA Kit (EMD Millipore, Billerica, MA, USA), respectively. Concentrations of glucose, triglycerides, and cholesterol were measured colorimetrically using Fluitest GLU, Fluitest TG, and Fluitest CHOL (Analyticon^®®^ Biotechnologies AG Am Mühlenberg 10, 35104 Lichtenfels, Germany), respectively. Concentrations of free fatty acids were measured using NEFA FS DiaSys kits (DiaSys Diagnostic Systems GmbH, Holzheim, Germany).

### 4.4. Expression and Purification of Mouse FGF21

The mouse FGF21 coding sequence (aa 29–210) was optimized for *E. coli* expression and synthesized by Genewiz (South Plainfield, NJ, USA). This DNA sequence was subcloned into the expression vector pE-SUMOpro (LifeSensors Inc., Malvern, PA, USA). This construct was used for the induction of the fusion 6xHis-SUMO-fgf21 protein in *E. coli* BL21 (DE3) cells. The purified 6xHis-SUMO-fgf21 was cleaved using SUMO protease 1 and loaded onto a column with Ni-NTA resin. The FGF21 protein (aa 29–210) was in the flow-through fractions. Size exclusion chromatography on a Superdex 200 10/300 GL column was used as a final purification step. The absence of bacterial endotoxins in the FGF21 protein sample was confirmed by LAL-test (<0.2 U/µg protein).

### 4.5. Relative Quantitation Real-Time PCR

Gene expression was measured using relative quantitation real-time PCR. The mRNA levels of the following genes were assessed: genes encoding transcription factors and enzymes related to lipogenesis (peroxisome proliferator-activated receptor gamma*, Pparg*; fatty acid synthase, *Fasn*; lipoprotein lipase, *Lpl*; acetyl-coenzyme A carboxylase alpha, *Acca*; acetyl-coenzyme A carboxylase beta, *Accb*; diglyceride acyltransferase, *Dgat*), lipolysis (adipocyte triglyceride lipase, *Pnpla2*; hormone-sensitive lipase, *Lipe*), fatty acid oxidation (peroxisome proliferator-activated receptor alpha, *Ppara*; peroxisome proliferator-activated receptor γ coactivator protein-1α, *Pgc1*; carnitine palmitoyltransferase 1a and 1b, *Cpt1α* and *Cpt1b*; uncoupling protein 3, *Ucp3*), thermogenesis (uncoupling protein 1, *Ucp1*; deiodinase, iodothyronine, type II, *Dio2*), gluconeogenesis (glucose-6-phosphatase, *G6p*; phosphoenolpyruvate carboxykinase 1, *Pck1*), and glycolysis (glucokinase, *Gck*; pyruvate kinase, *Pklr*); genes encoding glucose transporters (insulin-dependent glucose transporter 4, *Slc2a4*; insulin-independent glucose transporters 1 and 2, *Slc2a1* and *Slc2a2*); and genes related to the regulation of energy intake and expenditure (hypothalamic orexigenic neuropeptides Agouti-related protein, *Agrp*; neuropeptide Y, *Npy*; anorexigenic pro-opiomelanocortin, *Pomc*; corticotropin-releasing hormone, *Crh*).

Total RNA was isolated from tissue samples using an ExtractRNA kit (Evrogen, Moscow, Russia) according to the manufacturer’s instructions. First-strand cDNA was synthesized using Moloney murine leukemia virus (MMLV) reverse transcriptase (Evrogen, Moscow, Russia) and oligo(dT) as a primer. The TaqMan gene expression assays (Applied Biosystems, Foster City, CA, USA) listed in Table 4 were used for relative quantitative real-time PCR with β-actin as an endogenous control. Sequence amplification and fluorescence detection were performed on an Applied Biosystems ViiA 7 Real-Time PCR System. Relative quantification was performed using the comparative threshold cycle (CT) method.

### 4.6. Statistical Analysis

Each result is presented as an arithmetic mean ± SE for the sample size (i.e., number of mice) indicated. A repeated measures ANOVA with the factors “sex” (male, female), “experiment” (PBS, FGF21 administration), and “day of experiment” (7) was used to analyze FGF21 effects on food intake, total energy intake, the ratio of energy intake/BW, and body weight loss. A three-way ANOVA with factors “diet” (SD, HFD), “experiment” (PBS, FGF21 administration), and “day of experiment” (1–7) was used to analyze FGF21 effects on differential food intake between males and females; and a three-way ANOVA with factors “diet” “experiment”, and “sex” was used to analyze FGF21 effect on the proportion of energy consumed through SD and HFD. A two-way ANOVA with factors “sex” and “experiment” was used to analyze FGF21 effects on body weight, fat and lean masses, organ weight, blood parameters, and gene expression with multiple comparisons using the post hoc Newman–Keuls test. Results were considered significant at *p* < 0.05. The STATISTICA 6 software package (StatSoft, TIBCO Software Inc., Palo Alto, CA, USA) was used for analysis.

## 5. Conclusions

In female mice with diet-induced obesity, as well as in male mice, FGF21 administration beneficially affected glucose and lipid metabolism and taste preferences, despite significant sex differences in adiposity rates and metabolic characteristics. In both females and males, FGF21 inhibited the consumption of HFD, and this effect was associated with the inhibition of *Agrp* expression in the hypothalamus. The beneficial metabolic outcomes of FGF21 administration were associated with female-specific activation of *Pparg* and PPARg target genes in abdominal WAT. This preclinical study is the first to show that sex-specific metabolic features in mice with diet-induced obesity do not significantly affect the pharmacological effects of FGF21.

## Figures and Tables

**Table 1:** Weight of body and organs in male and female mice administered PBS or FGF21.

	Males		Females		*p* ANOVA
	PBS (*n* = 7)	FGF21 (*n* = 7)	PBS (*n* = 5)	FGF21 (*n* = 5)	
BW day 0 (g)	41.9 ± 0.6	43.0 ± 0.7	31.6 ± 0.4 ***	32.4 ± 1.5 ***	< 0.001, s
BW day 7 (g)	38.7 ± 0.7	38.1 ± 0.4	29.5 ± 0.5 ***	29.1 ± 1.6 ***	< 0.001, s
Fat day 0 (g)	15.5 ± 0.5	15.7 ± 0.5	10.1 ± 0.5 ***	11.2 ± 1.5 **	< 0.001, s
Fat index day 0	0.37 ± 0.01	0.364 ± 0.007	0.32 ± 0.01	0.34 ± 0.03	< 0.05, s
Fat day 7 (g)	12.7 ± 0.58	11.9 ± 0.4	8.2 ± 0.3 **	8.15 ± 1.53 **	< 0.001, s
Fat index day 7	0.33 ± 0.01	0.312 ± 0.008	0.28 ± 0.01	0.27 ± 0.03	< 0.05, s
Liver (g)	1.34 ± 0.06	1.38 ± 0.07	0.98 ± 0.02 ***	1.0 ± 0.04 ***	< 0.001, s
Liver index	0.035 ± 0.001	0.036 ± 0.002	0.033 ± 0.001	0.035 ± 0.002	NS
iBAT (g)	0.118 ± 0.007	0.114 ± 0.009	0.082 ± 0.004	0.083 ± 0.007	<0.001, s
iBAT index	(3.04 ± 0.18) × 10^−3^	(3.0 ± 0.24) × 10^−3^	(2.8 ± 0.14) × 10^−3^	(2.8 ± 0.11) × 10^−3^	NS

Data were analyzed with two-way ANOVA with factors: sex (s) and experiment; *** *p* < 0.001, ** *p* < 0.01 Newman–Keuls post hoc test.

**Table 2:** Biochemical blood parameters in male and female mice.

	Males		Females		*p* ANOVA
	PBS (*n* = 6)	FGF21 (*n* = 6)	PBS (*n* = 5)	FGF21 (*n* = 5)	
FFA (mM)	0.54 ± 0.18	0.63 ± 0.22	0.50 ± 0.11	0.49 ± 0.05	ns
Triglycerides (mM)	1.03 ± 0.05	0.92 ± 0.04	0.93 ± 0.08	0.91 ± 0.09	ns
Cholesterol (mM)	4.69 ± 0.26	3.53 ± 0.25 *	3.46 ± 0.18 ^+^	2.72 ± 0.09 *^+^	*p* < 0.001, s*p* < 0.001, e
Glucose (mM)	15.9 ± 1.0	13.7 ± 0.8	14.1 ± 0.9	11.6 ± 1.0	*p* = 0.05, s*p* < 0.05, e
Insulin (ng/mL)	6.9 ± 0.6	5.2 ± 0.5 *	3.5 ± 0.4 ^+^	1.8 ± 0.5 *^+^	*p* < 0.001, s*p* < 0.01, e
Adiponectin(mkg/mL)	4.9 ± 0.3	4.5 ± 0.3	6.5 ± 0.2 ^+^	6.1 ± 0.5 ^+^	*p* < 0.001, s
Leptin (ng/mL)	18.5 ± 3.9	14.3 ± 2.3	7.5 ± 0.9 ^+^	6.3 ± 1.7	*p* < 0.01, s

Data were analyzed with two-way ANOVA with factors “sex” (s) and “experiment” (e); * *p* < 0.05, FGF21 vs. PBS; ^+^ *p* < 0.05, males vs. females in PBS- or FGF21-treated mice, Newman–Keuls post hoc test.

**Table 3:** Experimental design.

Mouse Age (Weeks)		Experiment (Days)										
12–28	28–38	−2	−1	0	1	2	3	4	5	6	7	8
Obesity development (consumption of SD + HFD)	Selection of obese mice	Adaptation to Pheno-Master cages		Fat and lean mass	Injections of PBS or FGF21							Fat and lean mass atDecapitation
		Body weight, SD and HFD intake, locomotor activity										

**Table 4:** TaqMan Gene expression assays used for relative quantitation real-time PCR.

Protein	Gene	Gene Expression Assay ID
Acetyl-coenzyme A carboxylase alpha	*Acca*	Mm01304285_m1
Acetyl-coenzyme A carboxylase beta	*Accb*	Mm01204683_m1
Agouti related neuropeptide	*Agrp*	Mm00475829_g1
Beta-actin	*Actb*	Mm00607939_s1
Carnitine palmitoyltransferase 1a	*Cpt1a*	Mm01231183_m1
Carnitine palmitoyltransferase 1b	*Cpt1b*	Mm00487191_g1
Corticotropin releasing hormone	*Crh*	Mm01293920_s1
Diglyceride acyltransferase	*Dgat*	Mm00515643_m1
Deiodinase, iodothyronine, type II	*Dio2*	Mm00515664_m1
Fatty acid synthase	*Fasn*	Mm00662319_m1
Fibroblast growth factor 21	*Fgf21*	Mm00840165_g1
Glucose-6-phosphatase, catalytic	*G6pc*	Mm00839363_m1
Glucokinase	*Gck*	Mm00439129_m1
Insulin receptor	*Insr*	Mm01211875_m1
Leptin receptor	*Lepr*	Mm00440181_m1
Lipase, hormone sensitive	*Lipe*	Mm00495359_m1
Lipoprotein lipase	*Lpl*	Mm00434764_m1
Neuropeptide Y	*Npy*	Mm01410146_m1
Patatin-like phospholipase domain containing 2 (adipocyte triglyceride lipase, ATGL)	*Pnpla2*	Mm00503040_m1
Peroxisome proliferative activated receptor, gamma, coactivator 1 alpha	*Ppargc1a (Pgc1)*	Mm01208835_m1
Peroxisome proliferator activated receptor alpha	*Ppara*	Mm0040939_m1
Peroxisome proliferator activated receptor gamma	*Pparg*	Mm00440940_m1
Phosphoenolpyruvate carboxykinase 1, cytosolic	*Pck1*	Mm01247058_m1
Pro-opiomelanocortin	*Pomc*	Mm00435874_m1
Pyruvate kinase liver and red blood cell	*Pklr*	Mm00443090_m1
Solute carrier family 2 (facilitated glucose transporter), member 1 (GLUT1)	*Slc2a1*	Mm00441480_m1
Solute carrier family 2 (facilitated glucose transporter), member 2 (GLUT2)	*Slc2a2*	Mm00446229_m1
Solute carrier family 2 (facilitated glucose transporter), member 4 (GLUT4)	*Slc2a4*	Mm00436615_m1
Uncoupling protein 1 (mitochondrial, proton carrier)	*Ucp1*	Mm01244861_m1
Uncoupling protein 3 (mitochondrial, proton carrier)	*Ucp3*	Mm01163394_m1

## Data Availability

The data presented in this study are available on request from the corresponding author.
